# Cross-cultural adaptation and translation into Brazilian Portuguese of the instruments Sick Control One Stone Fat Food Questionnaire (SCOFF), Eating Disorder Examination Questionnaire (EDE-Q) and Clinical Impairment Assessment Questionnaire (CIA)

**DOI:** 10.1590/2237-6089-2019-0083

**Published:** 2020-10-08

**Authors:** Carolina Meira Moser, Luciana Terra, Andressa da Silva Behenck, Miriam Garcia Brunstein, Simone Hauck

**Affiliations:** 1 Programa de Transtornos Alimentares em Adultos Hospital de Clínicas de Porto Alegre Porto AlegreRS Brazil Programa de Transtornos Alimentares em Adultos, Hospital de Clínicas de Porto Alegre (HCPA), Porto Alegre, RS, Brazil.; 2 Departamento de Psiquiatria Universidade Federal do Rio Grande do Sul Porto AlegreRS Brazil Departamento de Psiquiatria, Universidade Federal do Rio Grande do Sul (UFRGS), Porto Alegre, RS, Brazil.; 3 Laboratório de Pesquisa em Psiquiatria Psicodinâmica Programa de Pós-Graduação em Psiquiatria e Ciências do Comportamento UFRGS Porto AlegreRS Brazil Laboratório de Pesquisa em Psiquiatria Psicodinâmica, Programa de Pós-Graduação em Psiquiatria e Ciências do Comportamento, UFRGS, Porto Alegre, RS, Brazil.

**Keywords:** SCOFF Questionnaire, Eating Disorder Examination Questionnaire, Clinical Impairment Assessment Questionnaire, translations, eating disorder, instruments

## Abstract

**Introduction:**

Eating disorders (EDs) affect up to 13% of young people and are associated with significant morbidity and mortality. Nevertheless, important, internationally recognized instruments for brief ED screening (Sick Control One Stone Fat Food Questionnaire [SCOFF]), symptom severity assessment and diagnosis (Eating Disorder Examination Questionnaire [EDE-Q]) and assessment of ED-associated psychosocial impairment (Clinical Impairment Assessment Questionnaire [CIA]) were not yet available in Brazilian Portuguese. Our objective was to perform the cross-cultural adaptation and translation into Brazilian Portuguese of the instruments SCOFF, EDE-Q and CIA.

**Method:**

The process involved a series of standardized steps, as well as discussions with experts. First, the relevance and adequacy of the scales’ items to our culture and population were extensively discussed. Then, two independent groups translated the original documents, creating versions that were compared. With the participation of external ED experts (i.e., who did not take part in the translation process), synthesized versions were produced. The syntheses were then applied to a focal group of patients with ED (n = 8). After that step, a preliminary version of the three scales in Brazilian Portuguese was produced and sent for back-translation by two English native speakers, who worked independently. A synthesis of the back-translations, along with the preliminary versions in Brazilian Portuguese, were sent to the original authors.

**Results:**

The Brazilian Portuguese versions of SCOFF, EDE-Q and CIA were approved by the original authors and are now available for use.

**Conclusion:**

This study provides important tools for the ED research field in Brazil.

## Introduction

Eating disorders (EDs) are considered serious mental illnesses that affect thousands of individuals worldwide regardless of age, ethnicity, skin color, nationality or gender. These conditions are associated with high personal, family and social costs.^1^

According to diagnostic criteria from the Diagnostic and Statistical Manual of Mental Disorders, 5th edition (DSM-5), EDs affect up to 13% of young women^2^ and are characterized by chronicity, relapse, functional impairment, future risk of obesity, depression, suicide attempts, anxiety disorders, psychoactive substance abuse and morbidity.^3 - 5^ Moreover, EDs have the highest mortality rate among all mental illnesses.^6^ Mortality risk is high even in individuals who have access to specialized treatment. Standardized mortality ratio ranges from 1.9 up to 6.2 (90 up to 520% more cases of death than in the general population).^7 , 8^ Despite the severity and impairments associated with EDs, only 20% of individuals with these disorders receive any treatment, which is partly due to an overall failure to recognize and identify cases.^9^

The psychopathology of ED can be divided into general and specific components. The general psychopathology consists of traits observed in many psychiatric disorders, the most common ones being depression and anxiety symptoms.^10^ Specific components include certain behaviors and attitudes characteristic of these disorders, such as extreme methods of controlling body weight or shape. Even though the measurement of general psychopathology poses no particular difficulty for either clinical or research purposes, since there are various instruments available, the evaluation of specific pathology in EDs is more problematic.^10^

Therefore, it seems relevant to have instruments for detecting ED cases and assessing ED psychopathology that do not require lengthy, expensive training of interviewers or clinical evaluation by specialists.^11^ In this sense, conducting judicious processes of cross-cultural adaptation and translation of internationally recognized scales for brief ED screening (Sick Control One Stone Fat Food Questionnaire [SCOFF]), diagnosis and symptom severity evaluation (Eating Disorder Examination Questionnaire [EDE-Q]), and assessment of ED-associated psychosocial impairment (Clinical Impairment Assessment Questionnaire [CIA]), is an essential contribution to the development of research into EDs in our country. Some isolated regional studies have been conducted on the prevalence of risk behaviors for EDs in Brazil, using screening instruments such as the Eating Attitudes Test (EAT),^12 - 15^ Bulimic Investigatory Test Edinburgh (BITE)^13^ and Disordered Eating Attitude Scale (DEAS).^12^ However, to the authors’ knowledge, approved Brazilian Portuguese versions of important scales such as SCOFF, EDE-Q and CIA were to date not available.

The objective of this study was to perform the cross-cultural adaptation and translation into Brazilian Portuguese of the instruments SCOFF, EDE-Q and CIA.

## Method

The study was approved by the research ethics committee of Hospital de Clínicas de Porto Alegre (CAEE 17889319.9.0000.5327).

The cross-cultural adaptation and translation process of SCOFF, EDE-Q and CIA to Brazilian Portuguese involved a series of standardized steps and discussions with experts. To ensure quality, all steps were conducted in compliance with both the International Society for Pharmacoeconomics and Outcomes Research (ISPOR) Task Force’s Principles of Good Practice for the Translation and Cultural Adaptation Process for Patient-Reported Outcomes^16^ and the European Regulatory Issues on Quality of Life Assessment Group (ERIQ-A)’s advice towards a multistep approach.^17^ Also, because no evidence was found in the literature in favor of one specific method, and because we believe that the quality of the translation and adaptation of any instrument is crucial to its proper use in both clinical and research settings, we have added some additional steps besides those described in the above-mentioned protocols. All steps followed in this study are described below.

Once authorization was obtained from the authors of the original instruments, the first step following our research group’s protocol was to gather a team of experts to extensively discuss the relevance and adequacy of the scales’ items to our culture and population. Afterwards, two independent groups (with four members each) comprised of psychiatrists and psychologists translated the original documents into Brazilian Portuguese, creating Brazilian Portuguese version 1 (BPV1) and Brazilian Portuguese version 2 (BPV2) of the three scales. This phase included discussions among the members of each group aiming at producing a Brazilian Portuguese version that could, on the one hand, preserve the original meaning, and on the other, take into account the characteristics of our own language and population, i.e., ensure that Brazilian Portuguese speakers would understand the sentences as intended. Subsequently, in order to refine the translations, both groups, along with two experts in ED who did not participate in the translation processes, compared BPV1 and BPV2, creating a Brazilian Portuguese version 3 (BPV3) for each of the three instruments. At this point, the BPV3s of EDE-Q and CIA were compared with the European Portuguese validated versions of these scales.^18^

**Figure 1:**
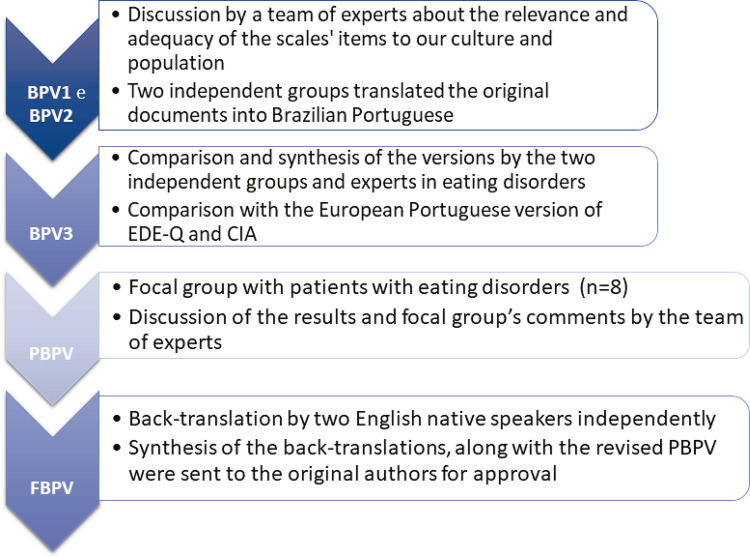
Flowchart of the cross-cultural adaptation and translation steps of the SCOFF, EDE-Q and CIA into Brazilian Portuguese. BPVx = Brazilian Portuguese version x; CIA = Clinical Impairment Assessment Questionnaire; EDE-Q = Eating Disorder Examination Questionnaire; FBPV = final Brazilian Portuguese version; PBPV = preliminary Brazilian Portuguese version; SCOFF = Sick Control One Stone Fat Food Questionnaire.

The BPV3s obtained for SCOFF, EDE-Q and CIA were then applied to a focal group of patients with EDs (n = 8) to assess their understanding of the questions and instructions contained in the translated questionnaires. Participants were encouraged to give suggestions to improve the questions/instructions in case of any unclear information. The patients of the focal group had no difficulty understanding the questions and instructions of the scales, but one of the eight patients suggested changes in the formatting of the EDE-Q scale responses. After that, the group of experts discussed the patients’ comments in order to further evaluate and potentially fine-tune the wording, bearing in mind both the need to retain the original meaning and also to find the best possible understanding for our population. However, the group concluded that the proposed changes in formatting were not relevant because they did not affect semantic equivalence and thus the layout of the EDE-Q was maintained unchanged, i.e., similar to the original instrument. As no changes were made at this stage, preliminary Brazilian Portuguese versions of the three scales were produced and sent for back-translation by two independent English native speakers who had no previous knowledge of the questionnaires or of the goals of our study. The back-translated versions were then compared and all items of the three scales were once again reviewed. A synthesis of the back-translations, along with the revised preliminary version of each instrument and the final report of all translation and cultural adaptation decisions were sent to the original authors for approval. After obtaining the original authors’ appraisal and approval of these versions, the final Brazilian Portuguese versions of the scales were considered adequate for use in clinical and research settings. Figure 1 summarizes all the steps involved in the translation and cross-cultural adaptation process ( Figure 1 ).

Below we present a brief description of the three instruments.

### Sick Control One Stone Fat Food (SCOFF) Questionnaire 19 , 20

The SCOFF is an ED screening questionnaire that comprises five yes/no questions. Each yes response equals 1 point; a score of 2 indicates a likely diagnosis of anorexia nervosa or bulimia nervosa.

### Eating Disorder Examination Questionnaire (EDE-Q ^10^ )

The EDE-Q is a 28-item self-report measure adapted from the Eating Disorder Examination (EDE), an investigator-based interview developed by Fairburn & Cooper.^21^ The EDE-Q focuses on the past 28 days and measures the core pathology of EDs, i.e., excessive importance of weight and shape in determining self-worth as well as frequency of core ED behaviors, including binge eating and compensatory behaviors. The instrument yields a global score as well as four subscale scores: eating concern, shape concern, weight concern and restraint. Of note, items addressing frequency of ED behaviors do not contribute to subscale or global scale scores. The EDE-Q is scored on a 7-point Likert scale, except for the items related to the frequency of behaviors, which are assessed in terms of the number of episodes occurring during the past four weeks. EDE-Q items in each subscale are summed and averaged to calculate the score, provided that more than half of the items within each respective subscale have been completed. The global scale score consists of the average of the four subscale scores. Higher scores are indicative of higher eating pathology.

### Clinical Impairment Assessment Questionnaire (CIA) ^22^

The CIA is a 16-item self-report measure developed to assess psychosocial impairment secondary to ED features, covering the past 28 days. It is designed for use immediately after the administration of the EDE-Q or other measure of current ED features. The items are scored on a 4-point Likert scale, which are summed to yield a single, global score that is indicative of the severity of secondary impairment as long as at least 12 of the items have responses.

## Results

The Brazilian Portuguese versions of SCOFF, EDE-Q and CIA were approved by the original authors and are available as online-only supplementary material.

## Discussion

This study provides the scientific community with internationally recognized instruments for ED screening (SCOFF), symptom severity and diagnosis (EDE-Q) and psychosocial impairment associated with the condition (CIA), now translated and adapted to Brazilian Portuguese through a very rigorous process. Therefore, it is our belief that it will contribute to ED research in Brazil.
