# Supplementary material for: Cross-cultural adaptation and translation into Brazilian Portuguese of the instruments Sick Control One Stone Fat Food Questionnaire (SCOFF), Eating Disorder Examination Questionnaire (EDE-Q) and Clinical Impairment Assessment Questionnaire (CIA)
Source: Trends Psychiatry Psychother. 2020 Oct 8;42(3):267–72. doi: 10.1590/2237-6089-2019-0083 (PMC7879072; doi:10.1590/2237-6089-2019-0083)
Supplement: Supplementary file 1 [file 2238-0019-trends-42-03-0267-suppl01.pdf]

http://dx.doi.org/10.1590/2237-6089-2019-0083

Sick Control One Stone Fat Food Questionnaire (SCOFF)

Questões SCOFF * Copyright Morgan JF, Reid F & Lacey JH, 1999

Por favor, responda às questões a seguir, assinalando SIM ou NÃO:

Você provoca **V**ômito por se sentir desconfortavelmente cheio (a)? **( ) SIM ( ) NÃO**

Você se preocupa em ter perdido o **C**ontrole sobre o quanto você come? **( ) SIM ( ) NÃO**

Você recentemente **P**erdeu mais de 6 kg em um período de 3 meses? **( ) SIM ( ) NÃO**

Você acredita estar **G**ordo (a) mesmo quando os outros dizem que você está muito magro (a)? **( ) SIM ( ) NÃO**

Você diria que a **C**omida domina sua vida? **( ) SIM ( ) NÃO**

* Cada “sim” equivale a 1 ponto; escore ≥ 2 indica probabilidade de anorexia nervosa ou bulimia nervosa

http://dx.doi.org/10.1590/2237-6089-2019-0083

Eating Disorder Examination Questionnaire (EDE-Q)

EATING QUESTIONNAIRE

Copyright Fairburn and Beglin, 2008

Questionário Alimentar

Instruções: As questões a seguir se referem apenas às últimas quatro semanas (28 dias). Por favor, leia cada questão cuidadosamente e responda todas as perguntas. Obrigado

Questões: 1 a 12: Por favor, circule o número apropriado à direita. Lembre que essas questões se referem apenas às últimas 4 semanas (28 dias).

	**Nos últimos 28 dias, em quantos dias ...**	**Nenhum dia**	**1-5 dias**	**6-12 dias**	**13-15 dias**	**16-22 dias**	**23-27 dias**	**Todos os dias**
**1**	Você <u>tentou</u> limitar intencionalmente (de propósito) a quantidade de comida que você come para influenciar sua forma corporal ou peso (tendo conseguido ou não)?	0	1	2	3	4	5	6
**2**	Você ficou longos períodos de tempo sem comer nada - 8horas ou mais, estando acordado (a)- para influenciar sua forma corporal ou peso?	0	1	2	3	4	5	6
**3**	Você <u>tentou</u> excluir da sua alimentação algum alimento que gosta para influenciar sua forma corporal ou peso (tendo conseguido ou não) ?	0	1	2	3	4	5	6
**4**	Você <u>tentou</u> seguir regras especificas em relação à sua alimentação (por exemplo, ter um limite máximo de calorias por dia) com o objetivo de influenciar sua forma corporal ou peso (tendo conseguido ou não)?	0	1	2	3	4	5	6
**5**	Você teve um desejo específico de ficar de estômago <u>vazio</u> com o objetivo de influenciar sua forma corporal ou peso?	0	1	2	3	4	5	6
**6**	Você teve um desejo específico de ter a barriga <u>totalmente reta</u> (chapada/"negativa")?	0	1	2	3	4	5	6
**7**	Ficar pensando em <u>comida, alimentação ou calorias</u>, tornou muito difícil se concentrar em coisas em que você tem interesse (por exemplo trabalhar, acompanhar uma conversa ou ler)?	0	1	2	3	4	5	6
**8**	Ficar pensando sobre <u>peso ou forma do corpo</u> tornou muito difícil se concentrar em coisas em que você tem interesse, (por exemplo, trabalhar, acompanhar uma conversa ou ler)?	0	1	2	3	4	5	6

**9**	Você teve um medo específico de perder o controle sobre a sua alimentação?	0	1	2	3	4	5	6
**10**	Você teve um medo específico de ganhar peso?	0	1	2	3	4	5	6
**11**	Você se sentiu gordo (a)?	0	1	2	3	4	5	6
**12**	Você teve um forte desejo de perder peso?	0	1	2	3	4	5	6

Questões 13 a 18: Por favor, preencha com o número apropriado nos campos à direita (pontilhado).
Lembre que as questões se referem apenas às últimas 4 semanas (28 dias).

Nas últimas quatro semanas (28 dias) ……

**13**	Nos últimos 28 dias, quantas <u>vezes</u> você comeu o que outras pessoas considerariam uma <u>quantidade exagerada de comida</u> (para aquela circunstâncias/ocasião)?	…………….
**14**	....Em quantas dessas vezes (que você respondeu na questão 13) você também teve a sensação de ter perdido o controle sobre a sua alimentação (no momento em que estava comendo)?	…………….
**15**	Nos últimos 28 dias, em quantos **<u>DIAS</u>** esses episódios de comer demais aconteceram (isto é, você comeu uma quantidade exagerada de comida <u>e</u> teve a sensação de ter perdido o controle naquele momento)?	…………….
**16**	Nos últimos 28 dias, quantas <u>vezes</u> você provocou vômito como uma maneira de controlar a sua forma ou peso?	…………….
**17**	Nos últimos 28 dias, quantas <u>vezes</u> você tomou laxantes como uma maneira de controlar a sua forma ou peso?	…………….
**18**	Nos últimos 28 dias, quantas <u>vezes</u> você se exercitou de uma forma “focada” ou “compulsiva” como uma maneira de controlar seu peso, forma ou quantidade de gordura, ou então para queimar calorias?	…………….

http://dx.doi.org/10.1590/2237-6089-2019-0083

Questões 19 a 21: Por favor, circule o número apropriado. <u>Por favor, observe que para essas questões o termo "compulsão alimentar" significa</u> comer o que outros considerariam uma quantidade exagerada de comida para as circunstâncias, acompanhada de uma sensação de ter perdido o controle sobre a alimentação naquele momento.

**19**	Nos últimos 28 dias, em quantos dias você comeu em segredo (escondido)? ....Não inclua os episódios de compulsão alimentar	Nenhum dia	1-5 Dias	6-12 dias	13-15 dias	16-22 dias	23-27 dias	Todos os dias
		0	1	2	3	4	5	6
**20**	Em quantas das vezes que você comeu, você se sentiu culpado (a) ou sentiu que tinha feito algo errado por causa dos efeitos que isso teria na sua forma ou peso? ....Não inclua os episódios de compulsão alimentar	Nenhuma das vezes	Em algumas vezes	Menos da metade	Metade das vezes	Mais da met ade	A maior parte das vezes	Todas as vezes
		0	1	2	3	4	5	6
**21**	Nos últimos 28 dias, o quão preocupado(a) você ficou com a ideia de as pessoas verem você comendo? ....Não inclua os episódios de compulsão alimentar	Nem um pouco		Leve mente		Moderada mente		Marcada mente
		0	1	2	3	4	5	6

Questões 22 a 28: Por favor, circule o número apropriado à direita. Lembre que essas questões se referem apenas às últimas 4 semanas (28 dias).

**Nos últimos 28 dias .....**	Nem um pouco		Levement e		Moderad amente		Marcada mente
**22** O seu <u>peso</u> teve influência na maneira como você se avalia/julga como pessoa?	0	1	2	3	4	5	6
**23** A sua <u>forma</u> teve influência na maneira como você se avalia/julga como pessoa?	0	1	2	3	4	5	6

**24**	Quanto você se sentiria chateado (a) se pedissem para você se pesar uma vez por semana (nem mais, nem menos) pelas próximas quatro semanas?	0	1	2	3	4	5	6
**25**	Quão insatisfeito (a) você tem estado com seu <u>peso</u>?	0	1	2	3	4	5	6
**26**	Quão insatisfeito (a) você tem estado com a sua <u>forma</u>?	0	1	2	3	4	5	6
**27**	Quão desconfortável você se sentiu vendo o seu corpo (por exemplo, vendo a sua forma no espelho, no reflexo de uma vitrine, enquanto tirava a roupa ou tomava banho)?	0	1	2	3	4	5	6
**28**	Quão desconfortável você se sentiu com <u>outras pessoas</u> vendo sua forma (por exemplo, em vestiários, na piscina/praia ou com roupas justas)?	0	1	2	3	4	5	6

Qual seu peso no momento? (Por favor, dê um valor aproximado) ....

Qual a sua altura? (Por favor, dê um valor aproximado) …………………

Se mulher: Nos últimos 3 a 4 meses você deixou de menstruar em algum ciclo? ....

Se sim, em quantos? ....

Você está tomando anticoncepcional? ………………

OBRIGADO

Clinical Impairment Assessment Questionnaire (CIA)

CLINICAL IMPAIRMENT ASSESSMENT QUESTIONNAIRE (CIA 3.0) Copyright Bohn and Fairburn, 2008
QUESTIONÁRIO DE AVALIAÇÃO DE PREJUÍZO CLÍNICO

INSTRUÇÕES

Por favor assinale com um "X" a coluna que melhor descreve como a sua vida tem sido afetada nas últimas 4 semanas (28 dias) por seus hábitos alimentares, exercícios ou sentimentos sobre sua alimentação, forma do corpo ou peso. Obrigado.

	**Nos últimos 28 dias em que medida os seus...**<br>**...hábitos alimentares**<br>**...exercícios**<br>**...ou sentimentos sobre comer, forma do corpo ou peso...**	**Nada**	**Um pouco**	**Bastante**	**Demais**
1	... dificultaram a sua concentração?				
2	... fizeram você se sentir crítico (a) consigo mesmo?				
3	... impediram você de sair com outras pessoas?				
4	... afetaram seu desempenho no trabalho (se aplicável)?				
5	... fizeram você ficar esquecido (a)?				
6	... afetaram sua capacidade para tomar decisões do dia-a-dia?				
7	... interferiram nas refeições com família ou amigos?				
8	... fizeram você ficar chateado (a)?				
9	... fizeram você sentir vergonha de si mesmo (a)?				
10	... tornaram difícil sair para comer com outras pessoas?				
11	... fizeram você se sentir culpado (a)?				
12	... interferiram em fazer coisas que você costumava gostar?				
13	... fizeram você ficar desligado (a) /fora do ar?				
14	... fizeram você se sentir um fracasso?				
15	... interferiram na sua relação com os outros?				
16	... fizeram você se sentir preocupado (a)?				
